# Identification of chalcone isomerase gene family in *Astragalus mongholicus* revealed genes regulating isoflavone synthesis

**DOI:** 10.3389/fpls.2025.1612434

**Published:** 2025-08-19

**Authors:** Zhen Wang, Panpan Wang, Xinxin Wang, Lingyang Kong, Jianhao Wu, Weichao Ren, Xiubo Liu, Wei Ma

**Affiliations:** ^1^ Pharmacy of College, Heilongjiang University of Chinese Medicine, Harbin, China; ^2^ Key Laboratory of Xinjiang Phytomedicine Resource and Utilization, Ministry of Education, School of Pharmacy, Shihezi University, Shihezi, China; ^3^ College of Jiamusi, Heilongjiang University of Chinese Medicine, Jiamusi, China

**Keywords:** *Astragalus mongholicus*, chalcone isomerase, expression pattern, prokaryotic expression, AsODN

## Abstract

*Astragalus mongholicus* (AM) is the original plant of the famous traditional Chinese medicine Astragali Radix, and its isoflavones are important bioactive substances with wide-ranging medicinal values. The chalcone isomerase (CHI) serves a pivotal function in flavonoid synthesis. However, the CHI gene family in AM has not yet been characterized and systematically analyzed. The present study identified a number of eight *AmCHI*s distributed on five chromosomes and classified them into four types. The evolutionary relationships, conserved motifs, gene structures, and *cis*-elements of *AmCHI*s are discussed. The transcriptome revealed the expression profiles of the *AmCHI* genes in roots, stems and leaves. In order to characterize *AmCHIs* function, recombinant proteins AmCHI3, AmCHI4 and AmCHI5 were expressed in *Escherichia coli*, and the enzyme activity assay showed that the typeI AmCHI4 could only catalyze naringenin chalcone to chalcone, and the typeII AmCHI3 catalyzed the conversion of naringenin chalcone to chalcone and of isoliquiritigenin to Liquiritigenin. Type III AmCHI5 lacked catalytic activity. In addition, gene suppression of AmCHI3 was carried out by using antisense oligodeoxynucleotides (AsODN). Transient gene silencing of *AmCHI3* decreased the contents of calycosin, calycosin-7-glucoside and formononetin. indicating that *AmCHI3* gene has a positive regulatory effect on the synthesis of isoflavonoids in AM. These results provide the data support for future elucidation of the regulatory mechanism of *CHI* in isoflavone biosynthesis.

## 1 Introduction

*Astragalus membranaceus* (Fisch.) Bge. var. *mongholicus* (Bge.) Hsiao (AM) is perennial herb of Leguminosae. It is widely utilized as a medicinal plant across the globe (Fu et al., 2014). The dried roots of AM, known as Radix Astragali (Huang Qi), have been used in China for more than 2000 years as a TCM to tonify Qi and raise Yang, consolidate the body surface to stop sweating, produce fluid, nourish the blood and exert an astringent effect on muscle soreness (Kim et al., 2003). Moreover, AM contains abundant bioactive substances, including polysaccharides, saponins, flavonoids, amino acids, fatty acids (FAs), and trace elements. Among these bioactive components, saponins, polysaccharides, and isoflavonoids have been extensively studied and clinically applied (Kiyohara et al., 2010). Modern pharmacology research has demonstrated that Radix Astragali possesses a range of pharmacological activities, including the ability to enhance immune function, exert anti-inflammatory, antitumor, antidiabetic, and other effects (Zheng et al., 2020). Moreover, Radix Astragali has a pivotal role in the clinical treatment of coronavirus disease (COVID-19) as a principal component of Huashibaidu granules, which can be utilized to treat mild, moderate, and severe cases of COVID-19 (Huang et al., 2021).

Chalcone isomerase (CHI, EC 5.5.1.6) is not only one of the earliest recognized enzymes but also a crucial rate-limiting enzyme in the flavonoid synthesis pathway (Gensheimer and Mushegian, 2004). The *CHI* gene have been reported in plants, fungi, and bacteria (Herles et al., 2004).In 1988, the CHI protein has been purified from soybean plants and enzymatically characterized. It has shown that CHI catalyzes the cyclization of 2′,4′,4- trihydroxychalcone (I) to 4′,7-dihydroxyflavanone (II) and that the conversion efficiency can be increased 36,000-fold using CHI enzyme as catalysts compared with the rate of isomerization of the product itself (Bednar and Hadcock, 1988). Moreover, the catalysis of CHI is pH-dependent, with a catalytic efficiency of only 50% at pH 6.0 and up to 90% at pH 7.5 (Boland and Wong, 1975). Studies on the catalytic mechanism of CHI have revealed that enzyme–substrate and enzyme–intermediate complexes are formed during the catalytic process. Moreover, CHI forms enzyme intermediates much more efficiently than the basal form compared with the relatively lower catalytic efficiency of the branched acidic translocator enzyme (Hur et al., 2004).

The *CHI* gene belongs to the supergene family, has species specificity in plants, and can be classified into four types (Yin et al., 2019). Type I proteins are predominantly found in angiosperms and can only catalyze the generation of naringenin in a 2S configuration using naringenin chalcone as a substrate (Wolf-Saxon et al., 2023). Type II proteins are mainly found in Fabaceae and involved in the synthesis of isoflavones in the flavonoid synthesis pathway. These proteins can use naringenin chalcone and 6′-deoxychalcone as substrates to produce naringenin and glycyrrhizin, respectively (Braune et al., 2016). Type III proteins are localized in plastids, the site of new FA biosynthesis in plant cells (Kaltenbach et al., 2018). Type IV arises when the secondary structure elements around the FA-binding pocket underwent additional changes during the evolution of CHI, which, in turn, gave rise to CHI-like proteins. The amino acids in these pockets can interconnect with the substrate through hydrogen bonding; however, as the key amino acids in the active site are not conserved, type IV CHI proteins cannot convert chalcone to flavonoid (Hartmann, 2007). Therefore, the type I and type II *CHI* genes encode catalytically active CHI proteins that can effectively produce naringin.

The *CHI* genes have now been cloned from hundreds of plants, such as *Arabidopsis thaliana*, *Zea mays*, *Glycine max*, *Medicago sativa*, and *Lotus corniculatus*, and their functions in regulating flavonoid biosynthesis have been widely studied (Li, 2014). A recent study has successfully completed the whole-genome sequencing of AM and mapped the biosynthetic pathways of key medicinal components such as calycosin-7-O-β-D-glucoside (Chen et al., 2023). These findings provide critical genomic data to underpin investigations into the molecular regulatory mechanisms governing isoflavonoid biosynthesis in AM. In this study, the *AmCHI* gene family was characterized and systematically bioinformatically analyzed. The gene functions of AmCHI4 and AmCHI5 were verified by prokaryotic expression and *in vitro* enzymatic reaction. In addition, overexpression transgenic hairy roots generated through *Agrobacterium rhizogenes* mediated transformation and antisense oligonucleotides (AsODN) gene silencing technology were validated *in vivo* for the positive regulation of isoflavonoids in AM by the type II *AmCHI3* gene. The results of this study elucidated the regulatory mechanism of *AmCHIs* in AM isoflavone biosynthesis and provided a theoretical basis and foundation for the subsequent use of CHI protein for isoflavone biosynthesis.

## 2 Materials and methods

### 2.1 Plant material

AM seeds used in this experiment were purchased from a professional traditional Chinese medicine market in Anguo (Heibei, China). Soilless cultivation was performed in a light incubator by using an improved Hoagland Modified nutrient Salts (Coolaber, China) under a light intensity of 2,000~2,500 lux, a diurnal temperature of 25 °C (16 h), a night temperature of 20 °C (8h). Healthy plants with consistent growth were selected when AM seedlings were 40 d old, and part of them were sampled for leaves, stems, and roots, which were rapidly frozen with liquid nitrogen after collection and then kept at -80°C for qRT-PCR experiments

### 2.2 Identification of *CHI* genes in AM

The AM genomic files used for the study were downloaded from the GPGD website (http://www.gpgenome.com/species/109). TBtools software was used to extract and convert all coding sequences in the AM genomic to protein sequences (Chen et al., 2020). Firstly, *CHIs* in AM were identified using a Simple Hidden Markov Model (HMM) Search function in TBtools software using HMM mapping of CHI structural domains downloaded from the Pfam database (PF16035, PF16036, and PF02431) (E-value <1 × 10^−10^). Second, the CHI proteins identified in *A. thaliana* and *G. max* were used for BLASTP (E-value <1 × 10^−5^) with AM protein sequences to further screen for CHIs. Finally, the final *AmCHIs* was obtained after removal of redundant sequences. The physicochemical properties of *AmCHIs* were performed using the ExPASy database (https://www.expasy.org/). Prediction of subcellular localization of AmCHI proteins using the online site Euk-mPLoc (http://www.csbio.sjtu.edu.cn/bioinf/plant/#).

### 2.3 Phylogenetic analysis of AmCHI protein sequences in AM

The ClustalX (Thompson et al., 2002) software was employed to conduct sequence alignment analyses of protein from AM, *A. thaliana*, *G. max*, *L. corniculatus*, *Solanum lycopersicum*, and *Oryza sativa* derived from the Phytozome (Goodstein et al., 2012). The neighbor-joining approach was employed for phylogenetic analysis using the MEGA (Kumar et al., 2018), and the bootstrap repetition number was 1,000. Landscaping the phylogenetic tree using the EvolView website (https://www.evolgenius.info/evolview-v3/#login).

### 2.4 Chromosomal localization and collinearity analysis

Genome sequences of *Cannabis sativa* (GCA_029168945.1), *S. lycopersicum* (GCA_000188115.4), and *Malus domestica* (GCF_002114115.1) were obtained from the NCBI database (https://www.ncbi.nlm.nih.gov/). The MCScanX Wrapper function in TBtools was used for gene collinearity analysis, and Advanced Circos was used for inter-and intraspecific collinearity visualization analysis and to label the chromosomal position of the *AmCHI* genes.

### 2.5 Analysis of *AmCHI* genes structure and *cis*-elements

The conserved motifs of AmCHI proteins were identified through the use of MEME Suite (version 5.5.0), with 10 motifs and the rest set as default parameters. Information on phylogenetic trees, motifs, introns, and exon regions was integrated and visualized using the TBtools. A DNA sequence 2000 bp upstream of the *AmCHIs* coding gene was extracted as promoter region for *cis*-element prediction using PlantCare database and visualized using TBtools software.

### 2.6 *AmCHIs* expression profiling and qRT-PCR analysis

Raw transcriptome data of AM seedlings at different tissues were obtained through sequencing. The number of reads mapped to each gene was calculated using FeatureCounts (v2.0.3) (Liao et al., 2014). The FPKM value of each gene was then calculated based on the gene length, and heatmaps were plotted using TBtools software.

Total RNA extraction from samples using RNA extraction kit. Synthesize the first strand cDNA using the NovoScript^®^Plus All-in-one 1st Strand cDNA Synthesis SuperMix (gDNA Purge) (Novoprotein, Shanghai, China) kit. The qRT-PCR system was configured using the StarLighter HP SYBR Green qPCR Mix (Universal) (Beijing Foreverstar Biotech), and upsampling assays were performed using the AriaMx System platform (Agilent Technologies, Hercules, CA, USA). The *18s* rRNA gene was employed as an internal reference. The samples were analyzed in triplicate, and the final expression of each *AmCHI* was determined using the 2^−ΔΔCT^ method (Livak and Schmittgen, 2001).

### 2.7 Prokaryotic expression and western blot analysis

*AmCHI3, AmCHI4, and AmCHI5* were seamlessly cloned into the pET28a vector carrying the MS Fastcloning MultiS kit plus (Msunflowers Biotech). The constructed recombinant plasmid and the empty vector were transformed into BL21(DE3) Chemically Competent Cell (Weidi Biotechnology). A selection of positive clones was cultivated in liquid medium of LB composition, containing kanamycin (50mg/L) to OD_600_ = 0.6, and 0.5 mmol/L isopropyl-betaD-thiogalactopyranoside (IPTG) was added. Expression was induced at 37 °C, 30 °C, 25 °C, 20 °C, and 16 °C for 8h, respectively. The induced bacterial solution was collected, and the proteins were extracted and purified by His tag protein purification kit. The protein samples were electrophoresed by SDS-PAGE. In order to further identify the protein of AmCHIs, the purified protein was analyzed by Western Blot (WB). The primary antibody utilized was Rabbit Anti-His tag antibody. The secondary antibody employed was Goat Anti-Rabbit IgG H&L/HRP, and the PVDF membrane was treated with BiossECL Plus WB Substrate kit (Bioss, Beijing, China). Finally, the Amersham Imager 600 (Cytiva, USA) was used for photo observation. The reagents used in the WB experiments were brought grom Boaosen Biotechnology Co., LTD (Beijing, China).

### 2.8 Functional analysis of AmCHI *in vitro*

The activity of the AmCHI enzyme was quantified using a purified recombinant AmCHI protein (10ug/15ug), tris-HCl buffer (1M, pH 7.6), and 40 mmol naringenin chalone or isoliquiritigenin (Sichuan Vicky Biotechnology Co., Ltd., Sichuan, China) as substrate in 200µl system. The reaction conditions of the system with naringenin chalcone as substrate were vortexed for 10s at room temperature, subsequently, the sample was extracted twice with ethyl acetate, after which the solvent was evaporated using a nitrogen blower. Residues were dissolved in methanol and analyzed by UPLC. The reaction time of the system with Isoliquiritigenin as substrate was 37°C for 1h, and the rest was the same as above. Denatured proteins was used in the control group. The mobile phase was composed of acetonitrile (A) and water (B). Naringenin chalcone and naringenin were detected using the following gradient procedure was employed: 0~30 min, 45% A. Liquiritigenin and isoliquiritigenin were detected using the following gradient procedure was employed: 0~20 min, 30~80% A; 20~25 min, 100% A. The detection wavelength was 270 nm.

### 2.9 Gene suppression in AM using antisense oligonucleotides

The sense oligonucleotides (sODN) sequence aligned to the *AmCHI3* gene fragment and the reverse complementary antisense oligonucleotides (AsODN) sequence were designed using the online software Soligo (https://sfold.wadsworth.org/cgi-bin/index.pl). In order to prevent the primer from being degraded by nucleases, it is necessary to perform thiol modification on three bases at each end of the primer. The sODN primer and AsODN primer for the *AmCHI3* gene were diluted to 5 µM using dd H2O, and then Astragalus seedlings grown for 14 d were clipped at the roots and inserted into the diluted primers, and incubated for 48 h. The samples treated with the sODN primer served as the control group. For each treatment, ten plants with uniform growth were selected, and the experiment was replicated three times. The leaves of the treated plants were collected, rapidly frozen in liquid nitrogen, and then stored at -80 °C for subsequent molecular experiments and metabolic assays.

### 2.10 Detection of secondary metabolites of isoflavones

The leaves of AM that had been treated with the antisense oligodeoxynucleotide (AsODN) and sense oligodeoxynucleotide (sODN) primers were completely ground into a fine powder in liquid nitrogen. Subsequently, the powdered sample was lyophilized using a freeze dryer. Precisely 100 mg of the powder was weighed out and then 1200 microliters of 70% methanol was added to it. The mixture was sonicated in an ice-water bath for one hour. The sample was centrifuged at 12,000 rpm for 10 minutes. Subsequently, the supernatant was carefully extracted and filtered through a 0.22-μm syringe-driven membrane filter for LC-MS analysis. The samples were detected by LCMS-9030 (SHIMADZU, Japan) equipped with a Ultimate^®^ UHPLC XB-C18 column (1.8μm, 2.1 by 100mm). The mobile phase consisted of water containing 0.1% formic acid (designated as A) and acetonitrile (designated as B). The following gradient procedure was employed: 0~2 min, 95~60% A; 2~8 min, 60~30% A; 9~10 min, 5% A; 10~11 min, 5~95% A; 11~14 min, 95% A. The flow rate was set at 0.3 ml/min, and the column temperature was maintained at 40°C. The isoflavone metabolites including calycosin (CA), calycosin-7-glucoside (CAG) and formononetin (FO) (Shanghai yuanye Bio-Technology).

## 3 Results

### 3.1 Identification and sequence analysis of *AmCHIs*

Eight AmCHI genes were found in the AM genomic sequence, and the detailed information and physicochemical properties of all encoded proteins were presented in **Supplementary Table 1**. They were named *AmCHI1*–*AmCHI8* based on their chromosomal locations. The proteins encoded by *AmCHIs* ranged from 177 (AmCHI1) to 498 (AmCHI2) amino acids and corresponding MWs ranged from 20020.76 (*AmCHI1*) to 54944.30 Da (*AmCHI2*), and their theoretical pIs ranged from 4.94 (AmCHI4) to 9.36 (AmCHI8). The subcellular localization results showed that AmCHI1, AmCHI2, AmCHI3, AmCHI5, AmCHI6, AmCHI7, AmCHI8 proteins were localized in the chloroplast, and AmCHI4 protein was localized in the cytoplasm.

### 3.2 Phylogenetic and classification analysis of *AmCHIs*

**Figure 1:**
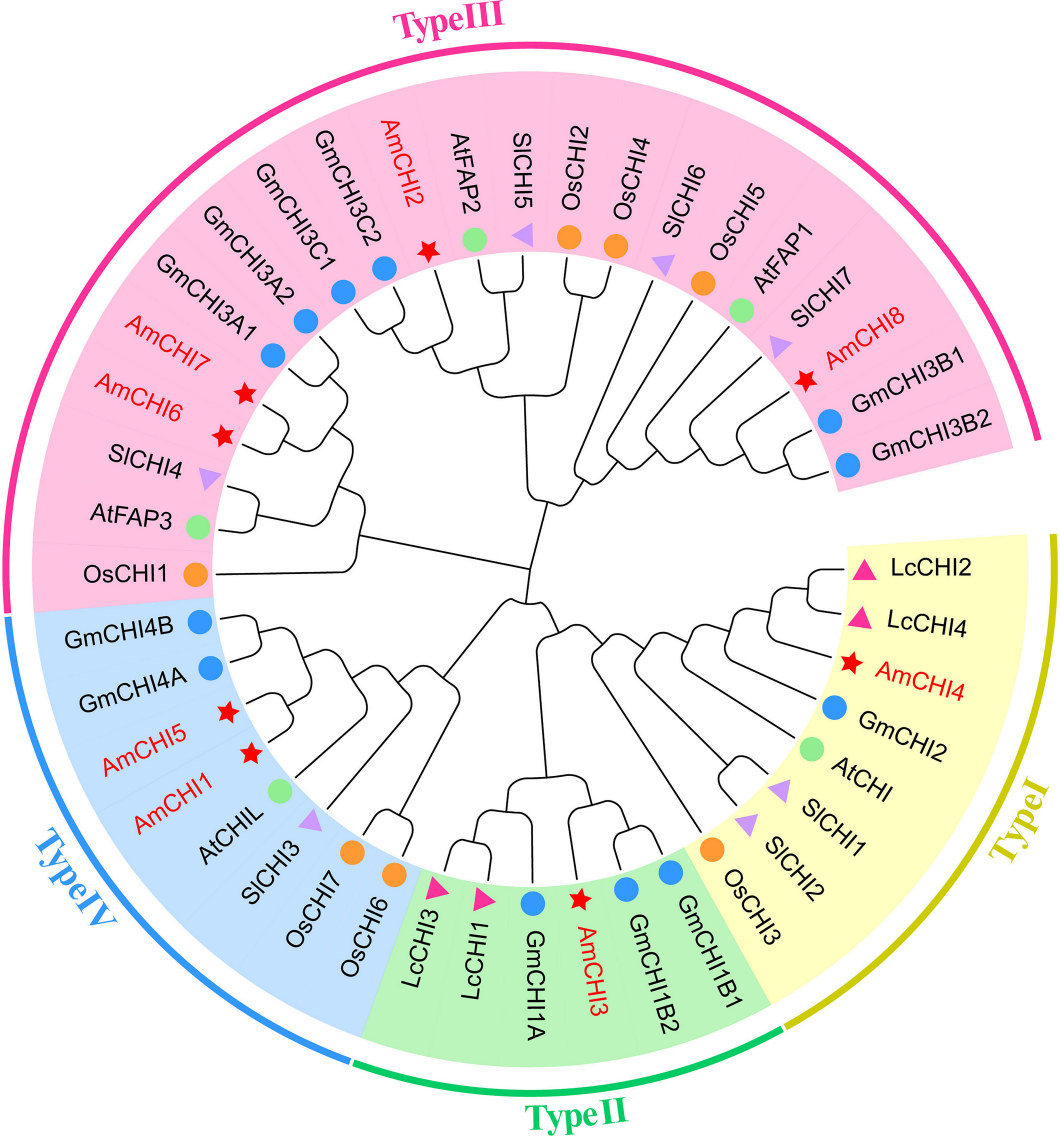
A phylogenetic tree depicting the CHI genes in AM and four additional plant species. The red star represents AM, the blue circle represents G. max, the orange circle represents O. sativa, the pink triangle represents L. corniculatus, the purple triangle represents S. lycopersicum , and the green circle represents A. thaliana. Different colors indicate different gene types.

To more comprehensively investigate the evolutionary origin and functional diversity of CHI genes in plants, this study constructed a phylogenetic tree using the Neighbor-Joining (NJ) method. (**Supplementary Table 2**). The results were consistent with the expectation that the *AmCHIs* could be categorized into four types (**Figure 1**). Types I and II had one each, type III had the most *AmCHI*s with four, and type IV had two. The *CHI* genes in AM were highly identical to those from *G. max* and *L. corniculatus*, which are also members of the Fabaceae family. Notably, *AmCHI3* was a unique type II gene in the Fabaceae family that may play a critical role in flavonoid biosynthesis in AM.

### 3.3 Chromosome locations and collinearity of *AmCHIs*

**Figure 2:**
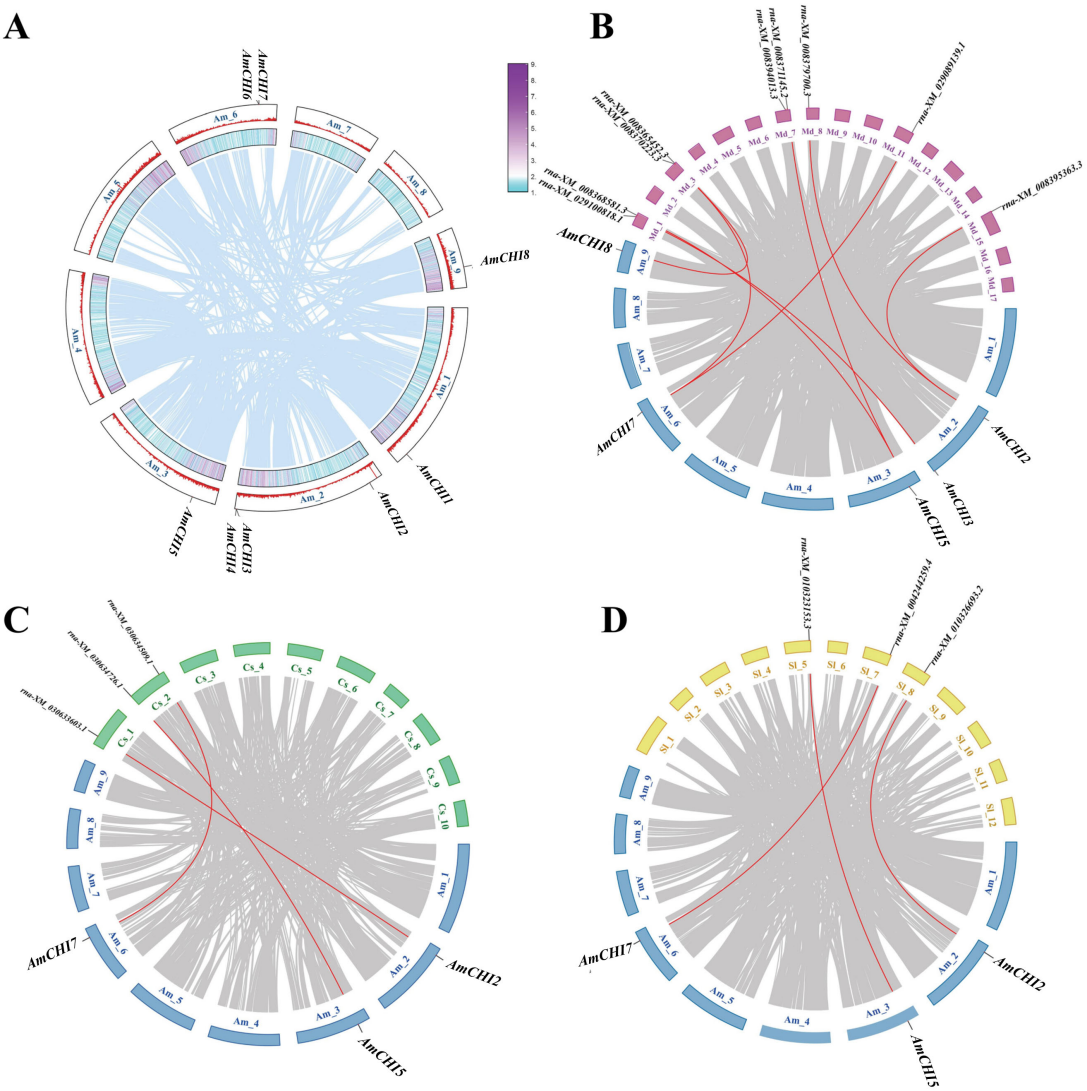
Chromosomal location and collinearity analysis of *AmCHI* genes. **(A)** Chromosome distribution and gene duplication events in *AmCHI* genes. **(B–D)** Gene collinearity between AM and other plants. The red lines represent *CHI* genes in homologous pairs.

Chromosomal localization maps were created based on information about the location of *AmCHI* genes on chromosomes of AM (**Figure 2**). The results showed that these eight *AmCHI* genes were distributed unevenly on 5 chromosomes. *AmCHI*s had the highest density of three on chromosome 2, followed by two on chromosome 6, and one on chromosomes 1, 3, and 9, respectively. No CHI gene was produced via segmental duplication in the *AmCHI* gene family (**Figure 2A**).

In this study, we investigated the genetic relationships and evolutionary trends of *AmCHI* family using collinearity analysis of AM, *M. domestica*, *C. sativa*, and *S. lycopersicum*. *M. domestica* had a closer kinship with AM, with five *AmCHI*s mapped to nine *MdCHI* genes (**Figure 2B**). In contrast, *C. sativa* and *S. lycopersicum* had only three *AmCHI*s mapped to the corresponding three *CsCHI*s and three *SlCHI*s (**Figure 2C**), which were identical (*AmCHI2*, *AmCHI5*, and *AmCHI7*) (**Figure 2D**). Therefore, it is possible that these genes have similar functions.

### 3.4 Identification of conserved motifs and analysis of gene structures in *AmCHIs*

**Figure 3:**
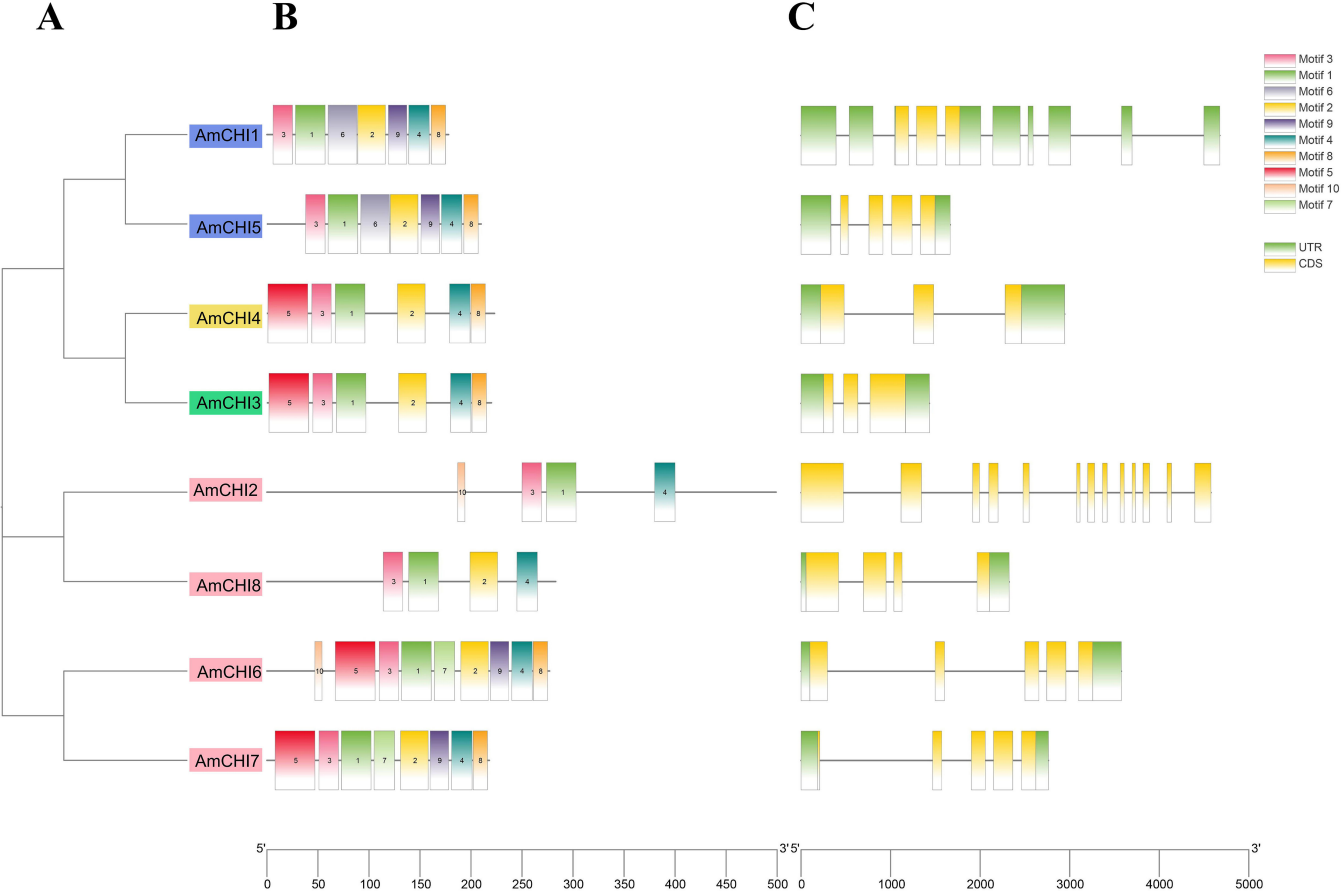
Phylogeny, conserved motifs, and gene structures of the *AmCHI* genes. **(A)** Phylogenetic tree of *AmCHIs*. **(B)** Conserved motifs. **(C)** Exon–intron structures of the *AmCHI* genes.

Conserved motifs are of paramount significance in the processes of gene family identification and classification. Ten motifs were found among the eight *AmCHIs* and named motif 1–motif 10. The results showed that each AmCHI contained 4–9 motifs with a width range of 8–30 amino acids (**Figure 3B**). Motif 1 and motif 3 were present in all heat shock factor proteins in *C. sativa*. The same types may have the same conserved motifs, and the composition of the conserved motifs in AmCHIs further supported the results of the phylogenetic classification (**Figure 3A**).

The structural diversity of the *CHIs* was revealed by analyzing the exon–intron structure of the *AmCHI* genes. The amount of introns in the *AmCHI*s varies from 2 to 12, with most *AmCHI* genes consisting of 2–4 introns (**Figure 3C**). Within the same type, most members had similar exon–intron numbers and arrangements, suggesting that closely related *AmCHI* genes may have similar structures.

### 3.5 Analysis of the *cis*-elements in *AmCHIs* promoters

**Figure 4:**
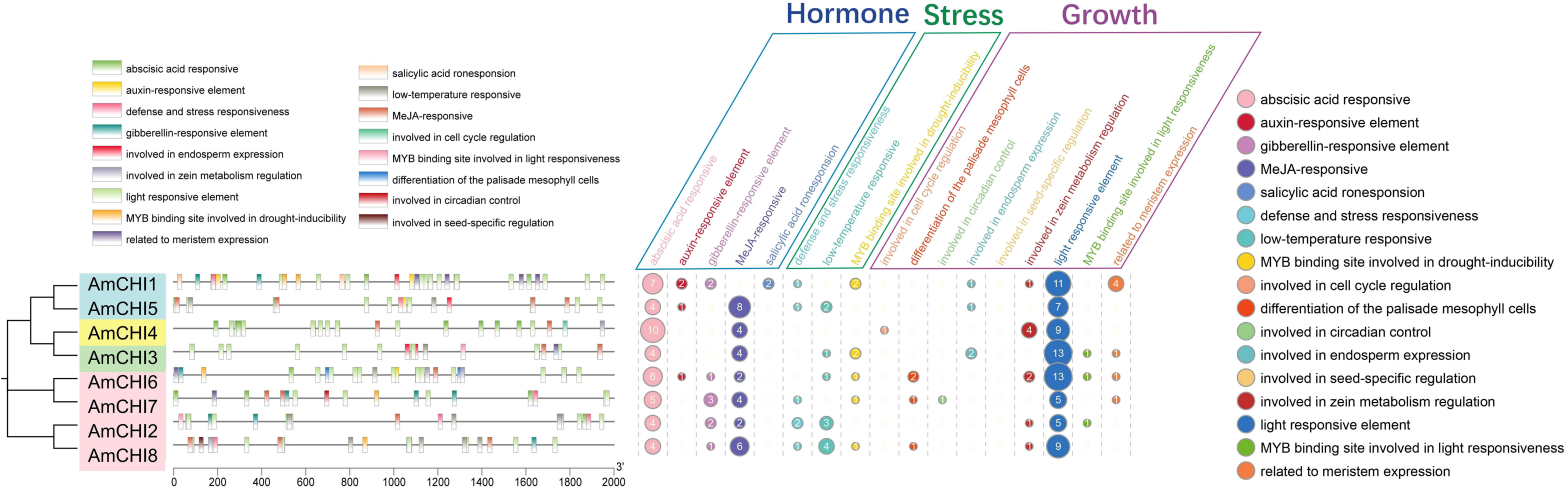
Promoter region *cis*-element prediction of *AmCHI* genes. Distinct colors are used to represent various *cis*-elements.

For investigating the potential functions of the eight *CHI*s in AM, we characterized and classified the *cis*-elements in promoter regions of these genes. The analysis revealed that a total of 209 *cis-*elements were screened from the promoter regions of all AmCHI genes. The identified *cis*-elements were classified into three functional categories, including nine growth-related, five hormone-related, and three stress-related responsive elements (**Figure 4**) (**Supplementary Table 3**). All eight *AmmCHI* genes contained at least one *cis*-element from these three categories, suggesting a critical role of the *CHI* gene family in all stages of AMM growth, development, and response to various stresses. We observed that the promoter regions contained binding sites for MYB transcription factors and were involved in the light and drought responses, suggesting that under certain circumstances, the *AmCHI* gene may be regulated by the *AmMYB* gene.

### 3.6 Analysis of the expression profile of the *AmCHIs* in different tissues and verification by qRT-PCR

**Figure 5:**
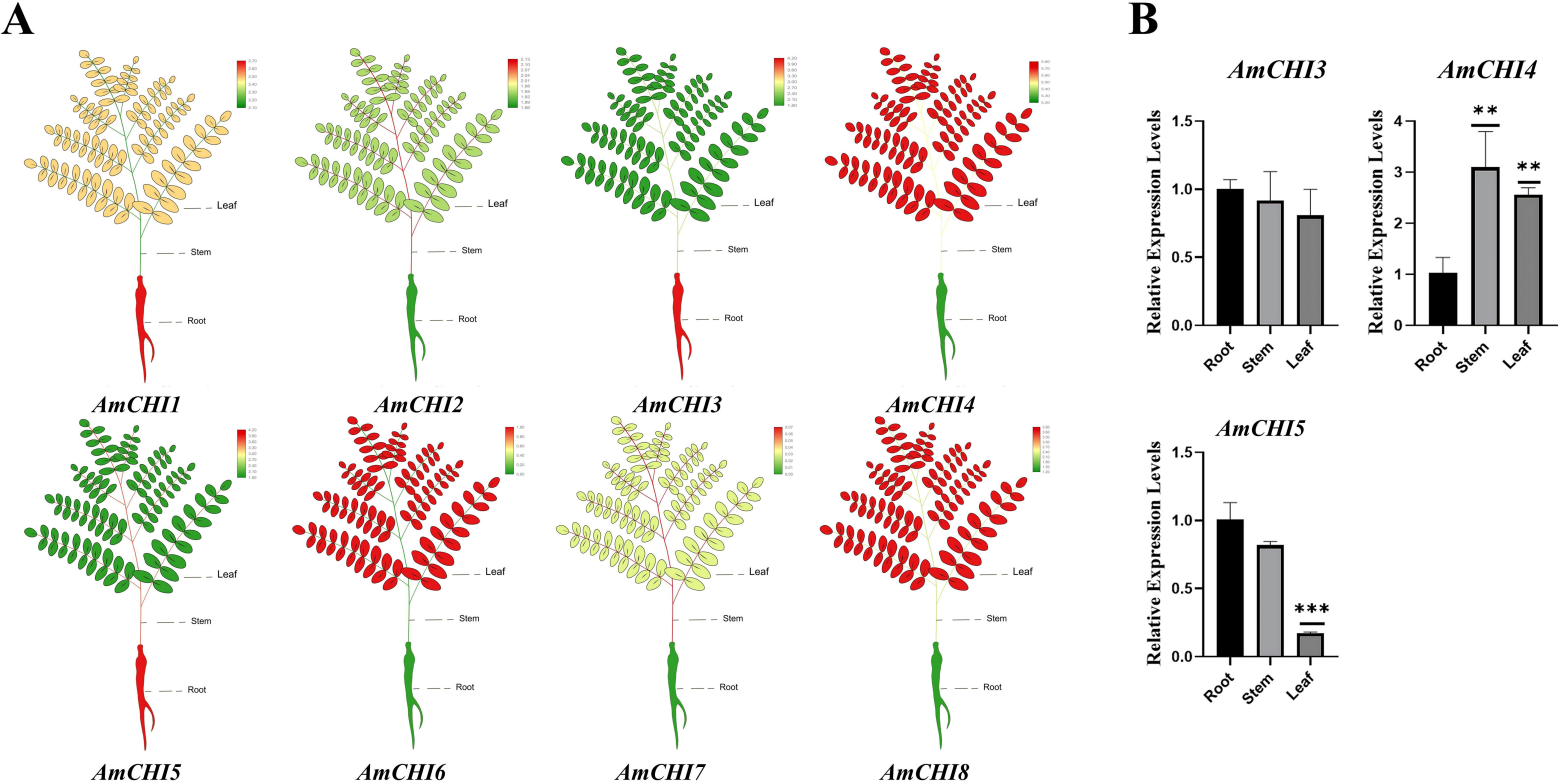
*AmCHI* genes expression profiling analysis. **(A)** Heatmap of the expression of the *AmCHI*s in AM leaves, stems, and roots. **(B)** The qRT-PCR was employed to detect the expression levels of *AmCHI* genes in different tissues of AM. Significance levels were denoted as **P < 0.01 and ***P < 0.001.

Gene expression pattern analysis can provide supporting evidence for mining gene functions. To elucidate the functions of *AmCHIs* during the seedling growth and secondary metabolism, we performed the transcriptome data from the seedling leaves, stems, and roots and extracted the FPKM values of all *CHI* genes (**Figure 5A**). Based on the heat map, *AmCHI2*, *AmCHI3*, *AmCHI4*, *AmCHI5*, and *AmCHI8* expressed in all tissues (FPKM>0.5) (**Supplementary Table 4**), with *AmCHI4* being the most highly expressed. Conversely, the *AmCHI7* transcript was not detected in any of the tissues. The *AmCHI6* gene showed an expression specific to leaf tissue. *AmCHI4* of type I and *AmCHI3* of type II, which may have catalytic functions, and *AmCHI5*, which is specifically expressed in roots, were selected for qRT-PCR to verify the reliability of the RNA-seq (**Figure 5B**) (**Supplementary Table 5**). The analysis indicated that the expression trends of the *AmCHI3*, *AmCHI4*, and *AmCHI5* in the leaves, stems, and roots of AM plants were consistent with that of the RNA-seq.

### 3.7 *In vitro* functional characterization of recombinant AmCHIs proteins

**Figure 6:**
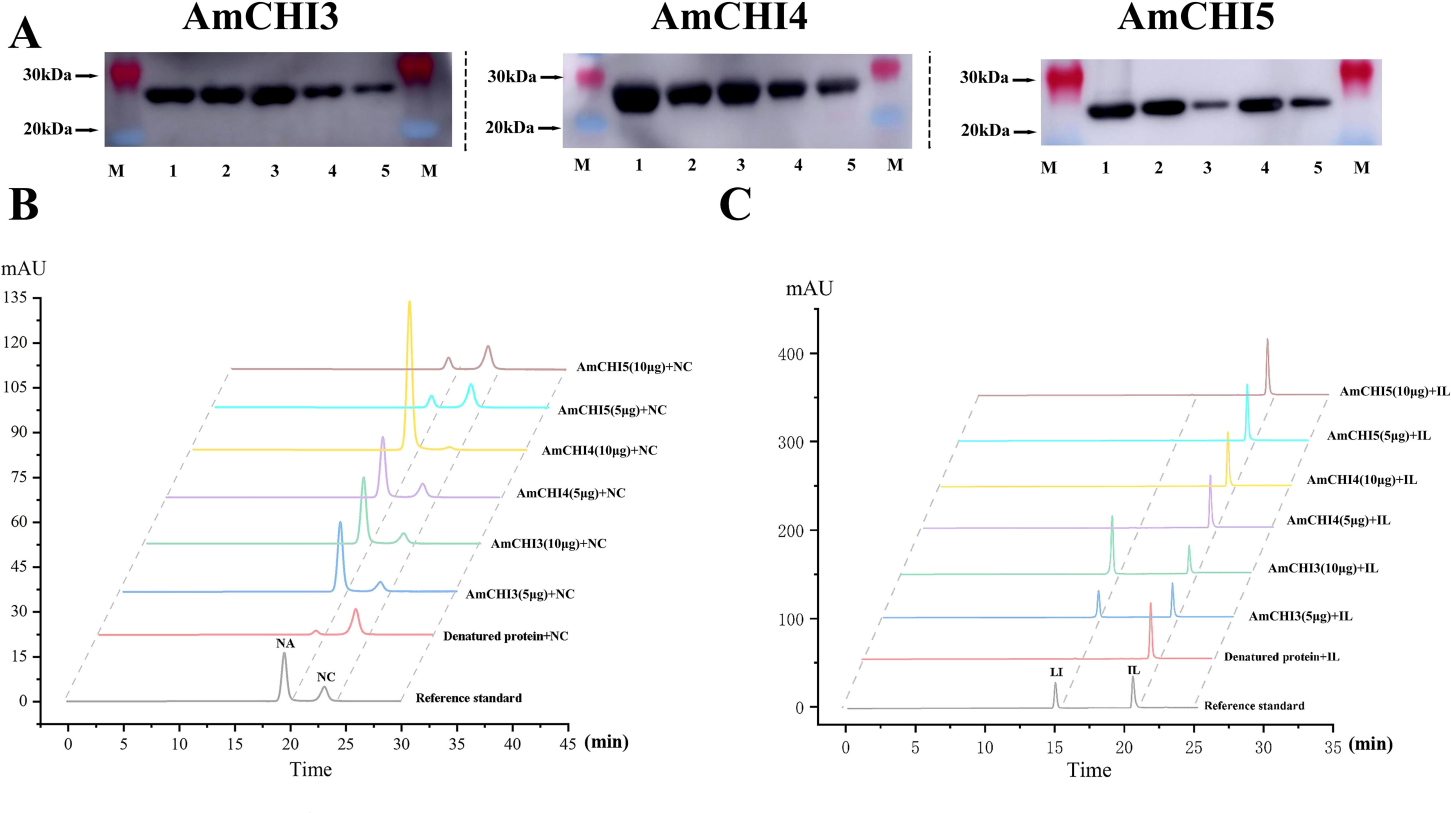
Recombinant AmCHIs proteins enzymatic assays. **(A)** Western blot detection of AmCHIs proteins. **(B)** Chromatogram of the *in vitro* reaction product of AmCHIs protein with naringenin chalcone as substrate. NA: naringenin; NC: naringenin chalcone. **(C)** Chromatogram of the *in vitro* reaction product of AmCHIs protein with isoliquiritigenin as substrate. IL, isoliquiritigenin; LI, Liquiritigenin.

To verify whether the *AmCHI3*, *AmCHI4* and *AmCHI5* genes encode functional CHI enzymes, recombinant AmCHI proteins were cloned from AM and inserted into a pET28a expression vectors with a His tag. Heterologous expression of recombinant proteins of AmCHI was attempted in BL21 (DE3) Chemically Competent Cell induced by 0.5 mM IPTG for 8 h at different temperatures (37, 30, 25, 20, and 16°C). The crude proteins were extracted and purified using a protein extraction kit. SDS-PAGE analysis showed that the crude protein extracted from *E. coli* had a clear, highly expressed protein at 23 kDa (**Supplementary Figure 1A**). After the purification of the crude protein, a clear single band at 23 kDa was observed (**Supplementary Figure 1B**), judged to be the recombinant AmCHI protein, and the size of the protein was in agreement with the predicted value. The recombinant AmCHI3 protein showed the highest expression at 30°C, and recombinant AmCHI4 and AmCHI5 showed the highest expression at 37°C. The recombinant AmCHI3, AmCHI4, and AmCHI5 proteins were expressed in soluble form in *E. coli*, and they were constitutively expressed at 37 °C with a small amount without IPTG induction. The analysis of the WB indicated that the product induced by the recombinant AmCHI expression strain exhibited His-tagged bands, detected using a His-specific antibody, at approximately 23 kDa, which is the band size of the expected the recombinant protein. (**Figure 6A**). The results showed that the AmCHI recombinant proteins had adequate reactogenicity and, thus, enzymatic activity compliant proteins were obtained.

In order to verify that AmCHI encodes functional CHI enzymes, purified AmCHI3, AmCHI4, and AmCHI5 proteins were used for *in vitro* enzymatic reactions. The findings indicated that Type I AmCHI4 solely catalyzes the conversion from naringenin chalcone to naringenin. Additionally, increasing the dosage of AmCHI4 can enhance the catalysis of a larger quantity of naringenin chalcone into naringenin. (**Figure 6B**). The type II AmCHI3 protein can not only catalyze the reaction of naringin chalcone to naringin, but also the reaction of isoliquiritigenin to Liquiritigenin. Increasing the protein content of AmCHI3 can promote the conversion of more isoliquiritigenin to Liquiritigenin. However, increasing the protein content of AmCHI3 cannot catalyze the conversion of naringin chalcone to more naringin (**Figure 6C**). The typeIII AmCHI5 protein does not have any catalytic function, which is consistent with previous reports.

### 3.8 Verification of *AmCHI3* genes expression and Isoflavone content by the AsODN transient system in AM leaves

**Figure 7:**
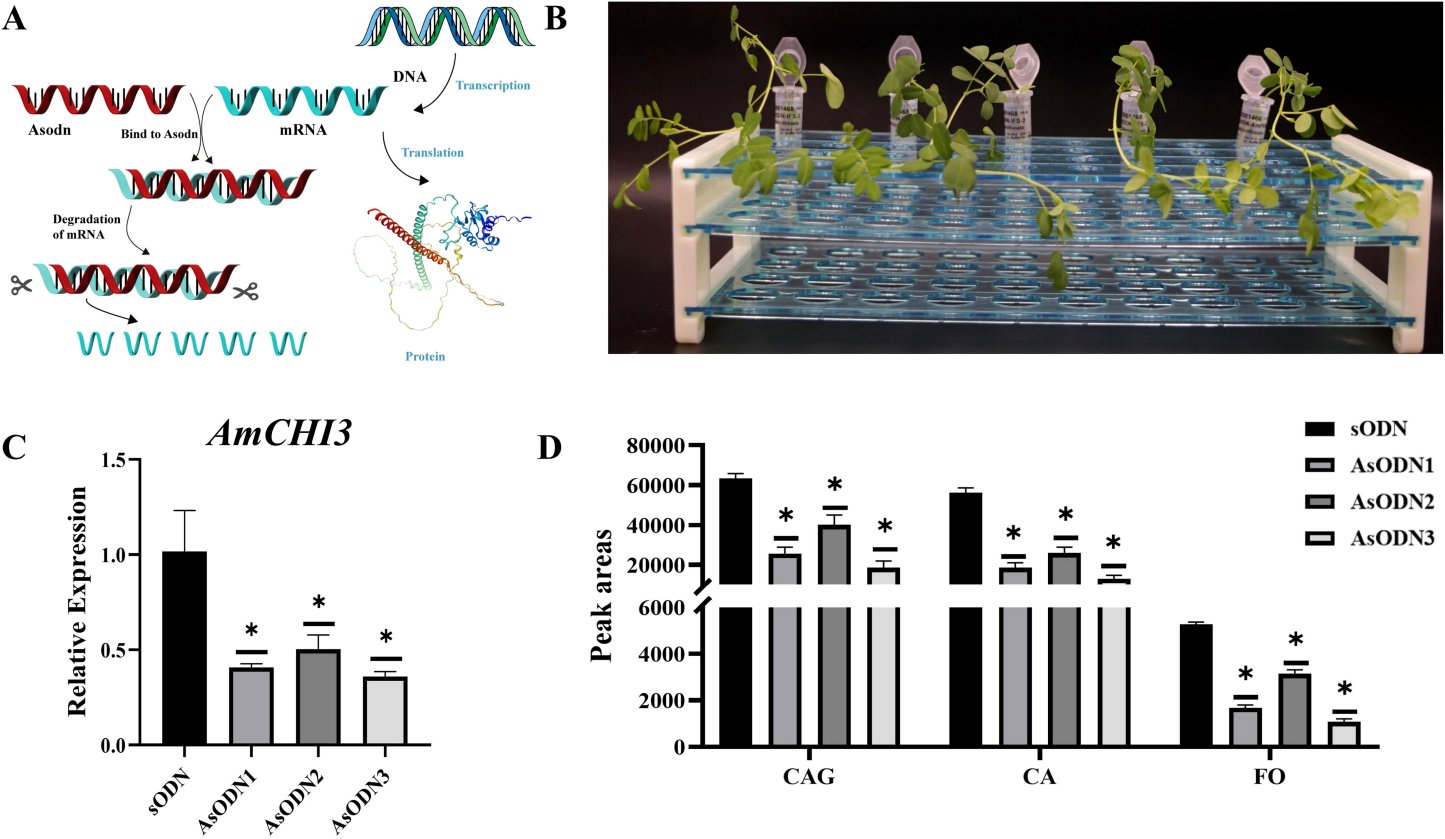
AsODN experiment of *AmCHI3* gene related to isoflavone metabolism in AM leaves. **(A)** Schematic diagram of AsODN technology principle. The protein image in the figure is sourced from the SWISS-MODEL website. **(B)** Schematic representation of the AsODN experiment. **(C, D)** The expression levels of *AmCHI3* gene and the relative changes in isoflavone content in plants treated with sODN primers and AsODN primers. AsODN1~3 indicates three biological replicates. Significance levels were denoted as *P <0.05.

AsODN is a technology to inhibit gene expression by specifically binding to the mRNA sequence of the target gene (**Figure 7A**). In this study, *AmCHI3* gene was tested (**Supplementary Table 6**). The secondary structure of the *AmCHI3* gene was analyzed by the online analysis software Soligo, and the optimal specific primers were designed based on the oligo binding energy (-6.4 kcal/mol) and nucleotide composition (50% GC). The primers were diluted to 5µM using ddH_2_O, and the root-cut AM seedlings were inserted into the primers, and samples were collected and tested after 2 d of incubation in the dark (**Figure 7B**). The results indicated that, when compared with the control (sODN), treatment with the AsODN primer led to a significant reduction in the expression level of the AmCHI3 gene (**Figure 7C**). Concurrently, the contents of CA, CAG and FO also decreased significantly (**Figure 7D**). This is contrary to the results of overexpression of *AmCHI3* gene, indicating that *AmCHI3* gene is a positively related enzyme gene for the synthesis of isoflavone compounds in AM.

## 4 Discussion

Flavonoids are of paramount importance in plant growth and development, plant resistance to adverse environmental conditions, as well as human nutrition and health. They are one of the three major secondary metabolites found in plants (Zhao et al., 2020). They comprise the main active ingredients in many TCM and are widely present in flowers, leaves, fruits, stems, seeds and roots used in certain Chinese medicines (Zouaoui et al., 2021). Flavonoids are crucial active chemical components of AM and have high medicinal and health values. However, the underlying mechanisms of flavonoid metabolism in AM remain largely elusive. Chalcone isomerase, a crucial rate-limiting enzyme in the flavonoid biosynthesis pathway, has been thoroughly explored at the molecular and biochemical levels. Nevertheless, the quantity and composition of CHI vary across different plant species (Corradini et al., 2011). The genomic date of AM has been published, providing the possibility of comprehensively investigating and studying the molecular evolution and function of the *CHI* family in AM. In the current study, we found eight *AmCHI*s in the AM, whereas there were only 5 *CHI*s in *A. thaliana* (Jiang et al., 2015), 5 in *Citrus grandis* (Wan et al., 2022), 4 in *Salvia miltiorrhiza* (Deng et al., 2018), 5 in *Dracaena cambodiana*, and 12 in *G. max* (Dastmalchi and Dhaubhadel, 2015). Phylogenetic analyses showed that the *AmCHI* genes represent all types of the *CHI* family, including type I (*AmCHI4*), which is uncommon in Fabaceae, type II (*AmCHI3*), predominantly found in Fabaceae, and types III (*AmCHI2*, *AmCHI6*, *AmCHI7*, and *AmCHI8*) and IV (*AmCHI1* and *AmCHI5*). Despite many *CHI* families and all their types being present in AM, based on the collinearity results, they were not generated through segmental duplication practices.

Plants produce large amounts of flavonoids as they grow, develop, and respond to various environmental factors (Shen et al., 2022), and the expression of the corresponding CHI genes is also affected. The flavonoid content of *D. cambodiana* increased when injected with NaCl and acetic acid, and the expression of the five *DcCHI* genes correlated positively (Zhu et al., 2016). Moreover, the expression of the *DcCHI1* and *DcCHI4* genes elevated significantly when *D. cambodiana* was subjected to mechanical damage, 6-benzyl aminopurine, and methyl jasmonate treatment (Zhu et al., 2021). Exposure to blue and red light significantly increased flavonoid content in tartary buckwheat seedlings, and the *FtCHI* gene positively correlated with flavonoid content, particularly under blue light (Zhang et al., 2019). These results indicate the potential of *CHI* to participate in various processes as plants grow and develop. In this study, we identified *cis*-elements associated with growth, development, hormones, and stress within the promoter regions of all AmCHI genes. These *cis*-elements encompass light-responsive elements, methyl jasmonate-responsive elements, as well as defense and stress responsive elements. This finding suggests that the *AmCHI* genes might respond to these factors. We found *cis*-elements in the promoter region of the *AmCHI* genes with MYB-binding sites in response to light and drought stress. *MYB* gene have been previously described to regulate CHI gene expression, such as the SmMYB1 transcription factor induced by methyl jasmonate, which increases anthocyanin biosynthesis in *S. miltiorrhiza* by activating the *CHI* gene and anthocyanin synthetase (Zhou et al., 2021). This suggests that there may be a possibility that the *AmMYB* gene regulates the *AmCHI* gene to increase flavonoid synthesis in AM.

Type I and type II CHI proteins exhibit enzymatic activities capable of catalyzing the stereospecific isomerization of chalcones, leading to the production of the corresponding flavonoids (Ni et al., 2020). Type I CHIs are ubiquitously found in vascular plants and play a crucial role in flavonoid synthesis (Wang et al., 2022). The type II *CHI* gene was earlier believed to be unique to the legume family and was considered responsible for isoflavone production (Ralston et al., 2005), whereas, in relatively more recent studies, this gene was also found to be present in ancient land plants (Cheng et al., 2018). In *O. sativa*, *OsCHI3* belongs to the type I *CHI* gene. The OsCHI3 protein has been expressed in *E. coli* and purified as a recombinant OsCHI3 protein. It has been demonstrated that OsCHI3 can catalyze the isomerization of naringin chalcone by detecting its catalytic activity (Park et al., 2021). The overexpression of the *CnCHI4* in Camellia nitidissima and *N. benthamiana* can increase their flavonoid contents, and *CnCHI4* belongs to the type I (Yu et al., 2022). The recombinant *L. japonicus CHI* family genes, *LjCHI1*, *LjCHI2*, and *LjCHI3*, have been expressed in *E. coli* and subjected to biochemical analyses. It was found that *LjCHI1* and *LjCHI3* are type II, receiving 6′-deoxychalcone and 6′-hydroxychalcone as substrates, whereas *LjCHI2* is the type I, only cyclizing 6′-hydroxychalcone, consistent with the classification results of the phylogenetic tree (Shimada et al., 2003). Type III CHIs belong to FA-binding proteins involved in FA metabolism that lack the catalytic ability to convert chalcones into flavonoids (Jez et al., 2000). In this study, we cloned the key types of *AmCHI3* and *AmCHI4* genes and the type III *AmCHI5* gene. We predicted the subcellular localization of the *AmCHIs* gene and found that it is expressed in chloroplasts. In *A. thaliana*, the three type III *CHI* genes, *FAP1*, *FAP2*, and *FAP3*, are located in the plastic stroma (Ngaki et al., 2012). We also successfully induced and purified recombinant AmCHI3, AmCHI4, and AmCHI5 in *E. coli*, the results of enzymatic reaction *in vitro* showed that the type I AmCHI4 protein could convert naringein chalcone to naringenin, the type II AmCHI3 protein could convert not only naringein chalcone to naringenin but also isoliquiritigenin to Liquiritigenin, and the type III AmCHI5 protein had no catalytic activity. These results not only validate the previous conclusions on the functional classification of CHI proteins, but also provide candidate genes for the subsequent biosynthesis of flavonoids.

Secondary metabolites are a group of small molecule organic compounds generated through plant secondary metabolism. Although they are non-essential for cellular life processes and plant growth and development, they possess diverse application values for humans (Zhu et al., 2023). Isoflavones are secondary metabolites mainly found in legumes, and AM, as a medicinal plant in the legume family, has isoflavonoids with important pharmacological values, such as CA, CAG and FO (Wang et al., 2023). Similarly, transient transformation systems applying AasODN for gene repression also can be used for validation of gene function and detection of secondary metabolites. AsODN is a technology that inhibits gene expression by binding specifically to the mRNA sequences of target genes, and it has been applied in many plants. For example, silencing of *CsANRa*, *CsANRb*, and *CsDFRa* genes by the AsODN transient silencing system significantly reduced anthocyanin content (Hui et al., 2022). In this study, gene suppression was performed using AsODN and transient gene silencing of the type II *AmCHI3* gene was achieved. The expression level of *AmCHI3* in the AmCHI3-AsODN strain was significantly down-regulated, while the contents of CA, CAG, and FO were significantly up-regulated, which confirmed that this gene positively regulates the synthesis of isoflavones in AM.

## 5 Conclusions

In this study, we performed genome-wide identification of the CHI gene family in AM and conducted systematic bioinformatics analyses. we cloned full-length transcripts of *AmCHI3*, *AmCHI4*, and *AmCHI5* genes. Enzyme activity assay indicated that typeI AmCHI4 protein could only catalyze naringenin chalcone to chalcone, and the typeII AmCHI3 protein not only catalyzed the conversion of naringenin chalcone to chalcone, but also catalyzed the conversion of isoliquiritigenin to Liquiritigenin. Type III AmCHI5 protein lacked catalytic activity. In addition, gene transient silencing experiments have demonstrated that the *AmCHI3* gene positively regulates the synthesis of isoflavones in AM. In summary, we identified the *AmCHI* gene family for the first time and determined their expression profiles and proteins, identifying candidate genes implicated in the biosynthesis of isoflavones in AM.

## Data Availability

The transcriptome data was deposited at NCBI database under accession number (PRJNA1064679).
