# Supplementary figures and images for: Identification of chalcone isomerase gene family in Astragalus mongholicus revealed genes regulating isoflavone synthesis
Source: Front Plant Sci. 2025 Aug 19;16:1612434. doi: 10.3389/fpls.2025.1612434 (PMC12401966; doi:10.3389/fpls.2025.1612434)

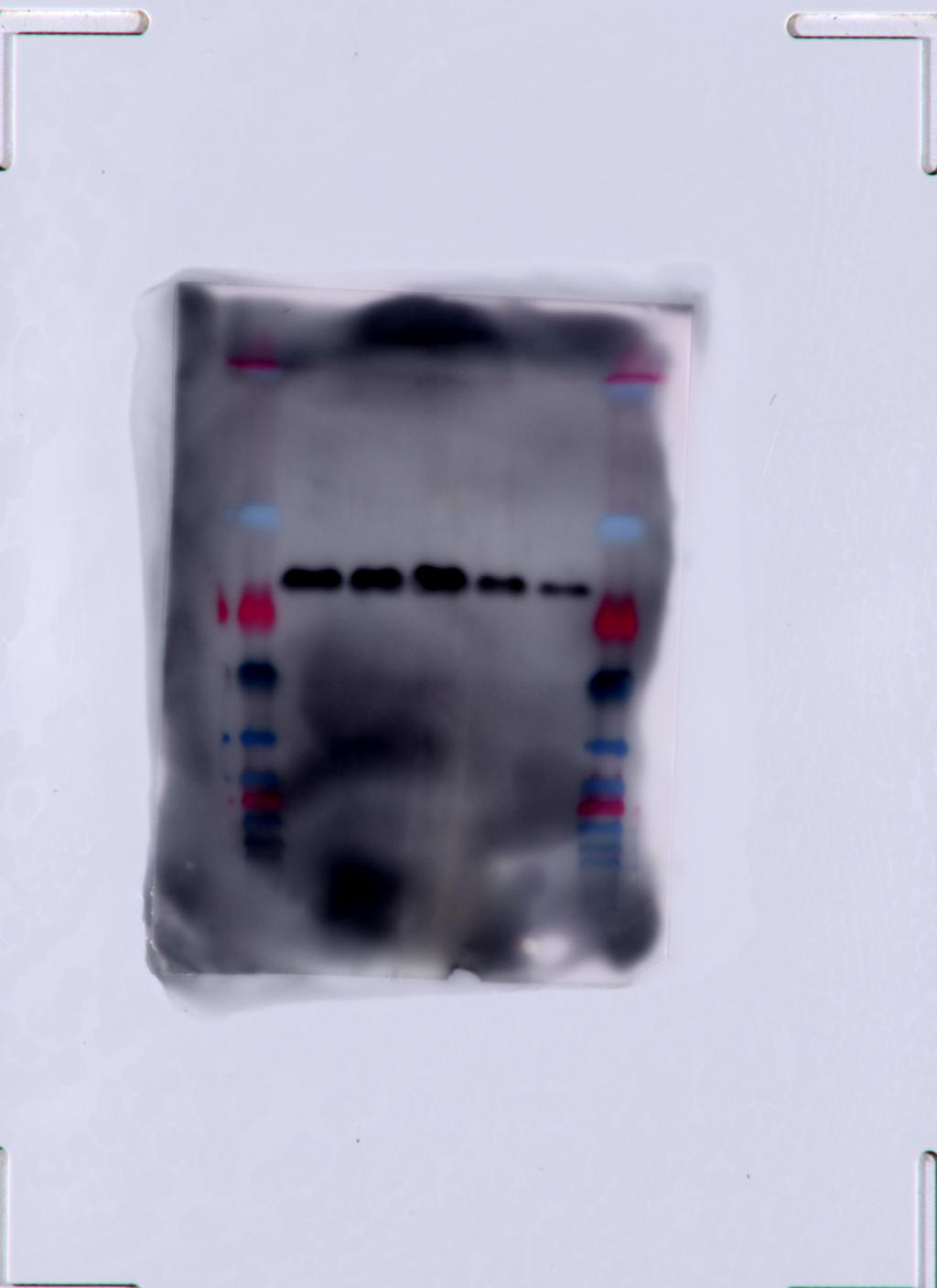

Supplement: Supplementary file 1 [file DataSheet1.zip › western blot images/3CHI-OriginalWesternBlots.tif]

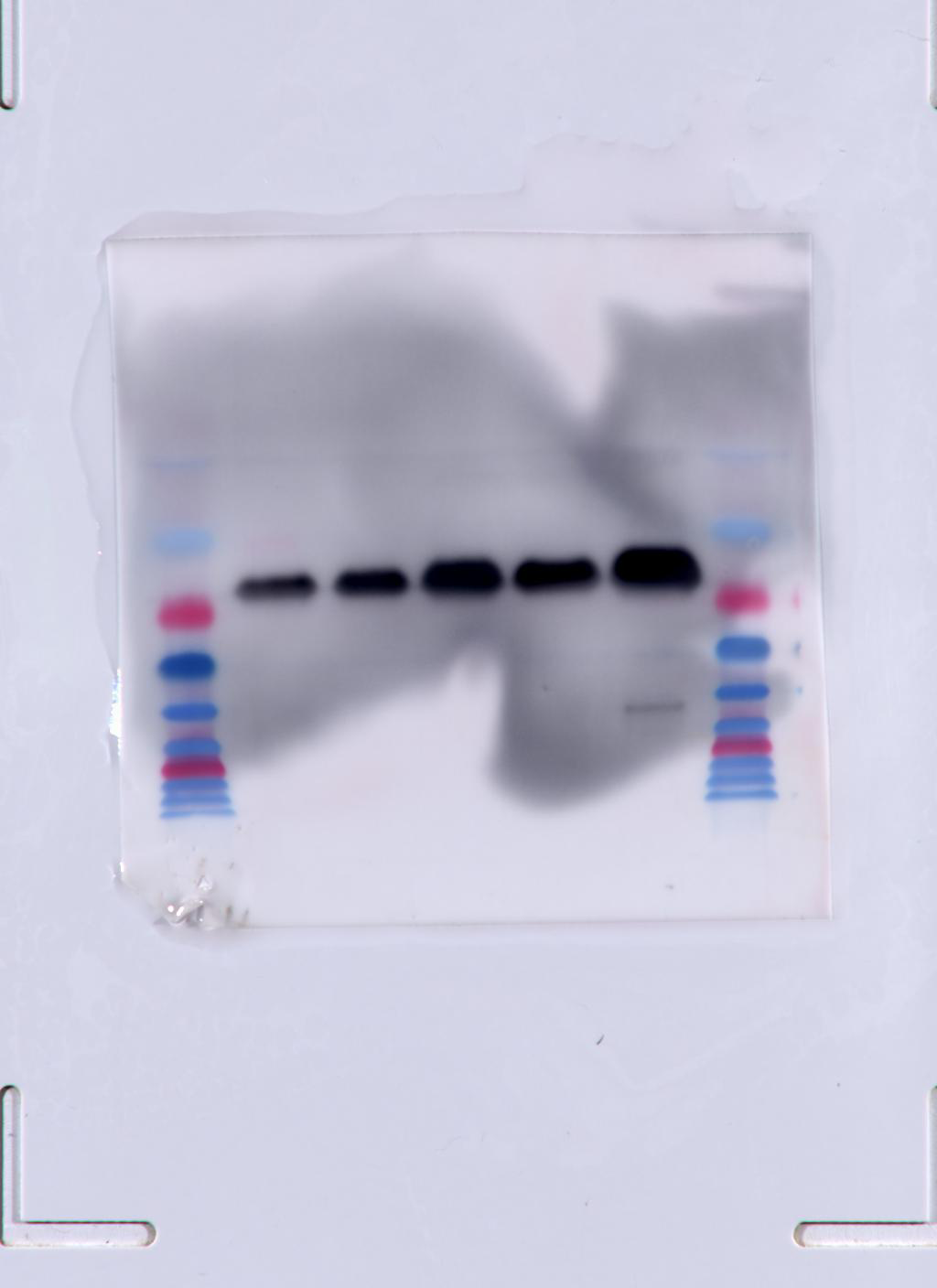

Supplement: Supplementary file 1 [file DataSheet1.zip › western blot images/4CHIOriginal Western Blots.tif]

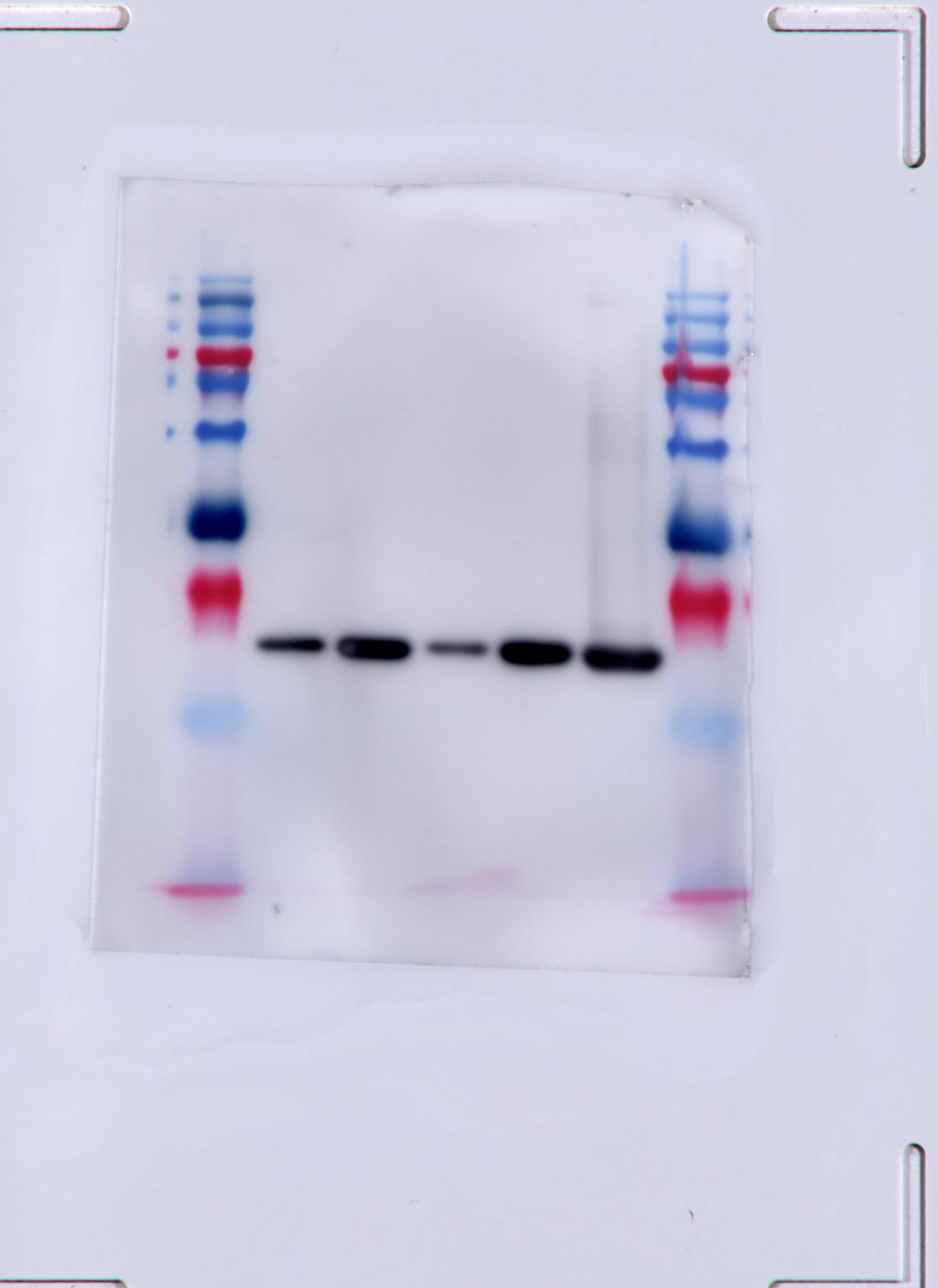

Supplement: Supplementary file 1 [file DataSheet1.zip › western blot images/5CHI-Original Western Blots.tif]
