# Supplementary material for: Identification of chalcone isomerase gene family in Astragalus mongholicus revealed genes regulating isoflavone synthesis
Source: Front Plant Sci. 2025 Aug 19;16:1612434. doi: 10.3389/fpls.2025.1612434 (PMC12401966; doi:10.3389/fpls.2025.1612434)
Supplement: Supplementary file 8 [file Table7.doc]

**
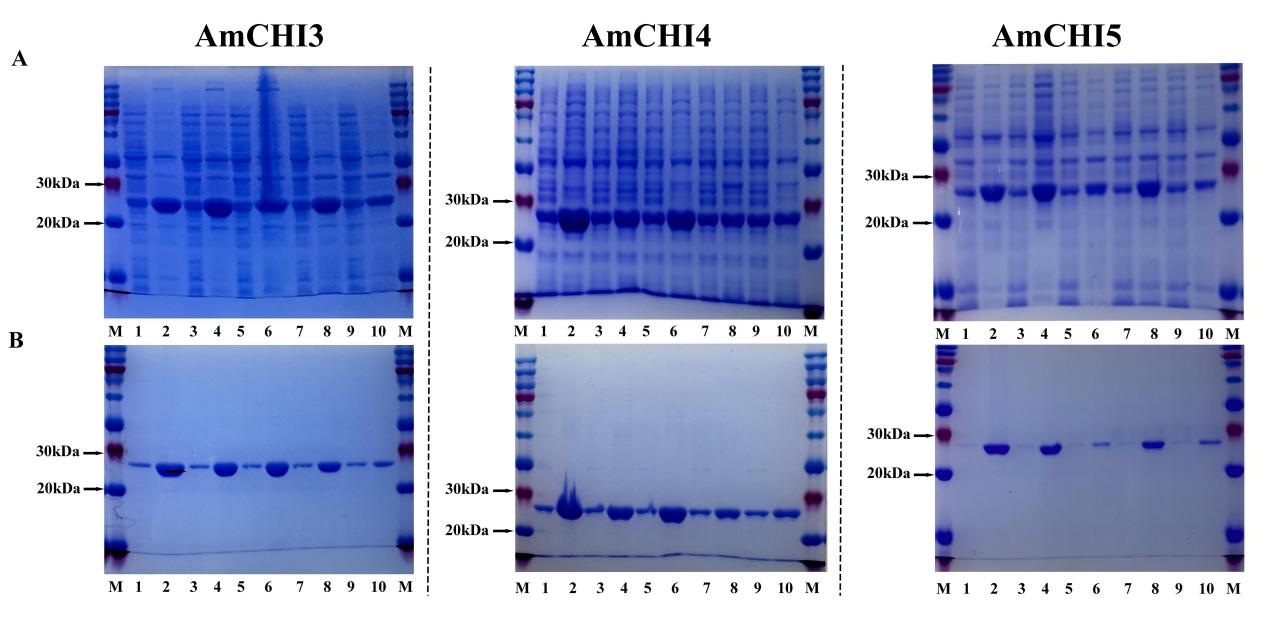
**

**Supplementary Fig. 1** SDS-PAGE and WB analyses of the prokaryotic expression product of AmCHI recombinant proteins. A: Expression of AmCHI3, AmCHI4, and AmCHI5 recombinant proteins in *E. coli*. M: Protein relative MW markers. Lanes 1, 3, 5, 7, and 9 show uninduced AmCHI recombinant protein cell lysate supernatant at 37 ℃, and lanes 2, 4, 6, 8, and 10 represent AmCHI recombinant protein cell lysate supernatant after induction at 37, 30, 25, 20, and 16 ℃, respectively, at 0.5 mM IPTG. B: Purification of recombinant proteins of AmCHI3, AmCHI4, and AmCHI5. Lanes 1, 3, 5, 7, and 9 show uninduced AmCHI recombinant proteins at 37 ℃, and lanes 2, 4, 6, 8, and 10 represent AmCHI recombinant proteins after induction at 37, 30, 25, 20, and 16 ℃, respectively, at 0.5 mM IPTG. C: Lanes 1, 2, 3, 4, and 5 represent AmCHI recombinant proteins after induction at 37, 30, 25, 20, and 16 ℃, respectively, at 0.5 mM IPTG.
